# Isolated Esophageal Langerhans Cell Histiocytosis in an Adult: A Case Report

**DOI:** 10.1002/ccr3.72484

**Published:** 2026-04-07

**Authors:** Jingxin Ye, Jing Hou, Zilong Ni, Mengmeng Ye, Feng Su

**Affiliations:** ^1^ Department of Gastroenterology, The Affiliated Suqian Hospital of Xuzhou Medical University Suqian Jiangsu China

**Keywords:** adult, case report, endoscopy, esophagus, Langerhans cell histiocytosis

## Abstract

Langerhans cell histiocytosis (LCH) is an inflammatory neoplastic disease characterized by the clonal proliferation of Langerhans cells, with a very low incidence, mostly in children. LCH in adults is extremely rare, with esophageal involvement being particularly uncommon. Its insidious onset and nonspecific manifestations often lead to diagnostic challenges and delayed diagnosis. Endoscopy and endoscopic biopsy in LCH patients with gastrointestinal symptoms are important for diagnosis, monitoring, and treatment. During histopathological diagnosis, special attention should be paid to the differential diagnosis of LCH from common gastrointestinal malignancies. Herein, we report a rare case of isolated esophageal LCH in a 34‐year‐old adult, who underwent endoscopic resection with no recurrence during follow‐up.

## Key Clinical Message

Isolated esophageal Langerhans cell histiocytosis in adults is extremely rare and often presents with nonspecific symptoms, making diagnosis challenging. Endoscopic biopsy with immunohistochemical staining for CD1a and S‐100 is essential for accurate diagnosis. Localized lesions can be effectively managed with endoscopic resection, and long‐term follow‐up is recommended to monitor for recurrence or systemic progression.

**Abbreviations**
CT: computed tomography
ESD: endoscopic submucosal dissection
LCH: Langerhans cell histiocytosis
PET: positron‐emission tomography
SS‐LCH: single‐system Langerhans cell histiocytosis

## 1 Introduction

LCH is a rare neoplastic disorder arising from the clonal proliferation of bone marrow‐derived dendritic cells, with an incidence of approximately 6 cases per million in children under 15 years and even lower prevalence in adults [1]. The adult prevalence rate is about 30% of that of children, with an annual incidence of only 1–1.5 cases per million [2, 3]. The clinical manifestations of this disease are diverse and can involve multiple organs and systems throughout the body [4]. While the exact pathogenesis of LCH remains incompletely understood, driver mutations have been identified in the majority of cases. More than half harbor the *BRAF‐*V600E mutation. In the remaining *BRAF*‐wildtype cases, other genetic alterations within the *MAPK* signaling pathway (RAS–RAF–MEK–ERK) are frequently implicated, including mutations in *MAP2K1, MAP3K1, ARAF, NRAS*, and *KRAS* [5, 6, 7, 8].

In adults with LCH bone is the most common site of involvement followed by skin, lymph nodes, and lungs with nonspecific clinical manifestations such as bone pain, skin lesions, cough, nausea, headache, weight loss, fever, and neuropsychiatric symptoms [9]. Gastrointestinal tract involvement is exceedingly rare, particularly isolated esophageal disease. Herein, we report a rare case of isolated esophageal LCH in a 34‐year‐old man who presented with nonspecific epigastric discomfort and was successfully managed with endoscopic submucosal dissection. This case highlights the diagnostic challenges of LCH at unusual sites and the importance of histopathological evaluation with immunohistochemical staining.

## 2 Case Presentation

**FIGURE 1:**
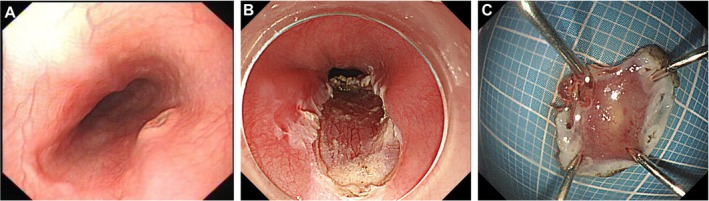
Endoscopic findings of isolated esophageal Langerhans cell histiocytosis. (A) Gastroscopy revealed a 5‐mm yellowish flat elevated lesion on the anterior wall of the esophagus, 30 cm from the incisors. (B) Endoscopic submucosal dissection (ESD) was performed to completely resect the lesion. (C) Postoperative endoscopic image showing the mucosal defect after ESD, with no residual lesion or complications.

A 34‐year‐old male patient was admitted to the hospital with recurrent epigastric vague pain and discomfort for more than six months. The patient had episodes of epigastric pain with no obvious cause, intermittent onset, no significant radiating pain, accompanied by occasional postprandial fullness and abdominal distension, and no gastrointestinal symptoms, such as nausea, vomiting, hematemesis, and melena, etc., therefore, he underwent upper‐GI endoscopy, which revealed a 5‐mm yellowish flat elevated lesion on the anterior wall of the esophagus, approximately 30 cm from the incisors (Figure 1A). Biopsy samples were obtained from this lesion, and histopathological examination showed abnormal proliferation of histiocytes with grooved nuclei and absent nucleoli, raising suspicion of Langerhans cell histiocytosis (Figure 3A).

**FIGURE 2:**
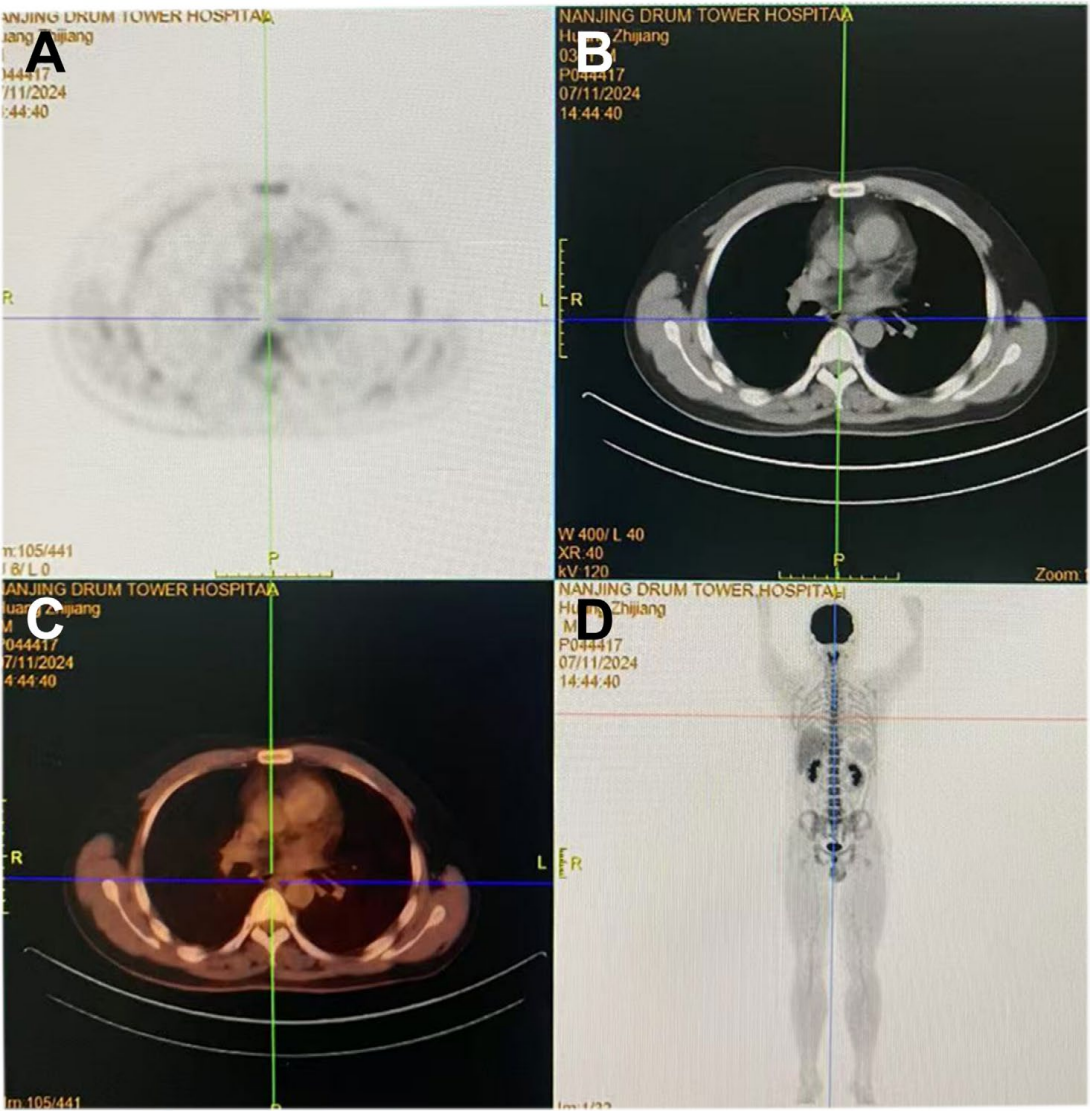
Positron‐emission tomography/computed tomography images of the patient. (A, B) Cross‐sectional computed tomography (CT) image of the esophagus area. (C) Cross‐sectional functional imaging images of the esophagus area. (D) Coronal functional imaging images of the esophagus area.

After hospitalization, the patient underwent further examinations, including PET/CT imaging (Figure 2), which revealed a localized slightly rough wall in the middle part of the esophagus, with no increase in radioactivity uptake. Several lymph nodes were seen in the cervical region bilaterally, the largest of which was about 1.3 × 0.7 cm, with increased radioactivity uptake, SUVmax = 4.4. Laboratory examinations: alpha‐fetoprotein (AFP), Carcinoembryonic antigen (CEA), Carbohydrate antigen 19–9 (CA199), CA50, CA242, CA724, thyroid function, routine blood tests, liver and kidney function, and coagulation test results were normal. Subsequently, endoscopic submucosal dissection (ESD) was performed for lesion removal (Figure 1B,C).

**FIGURE 3:**
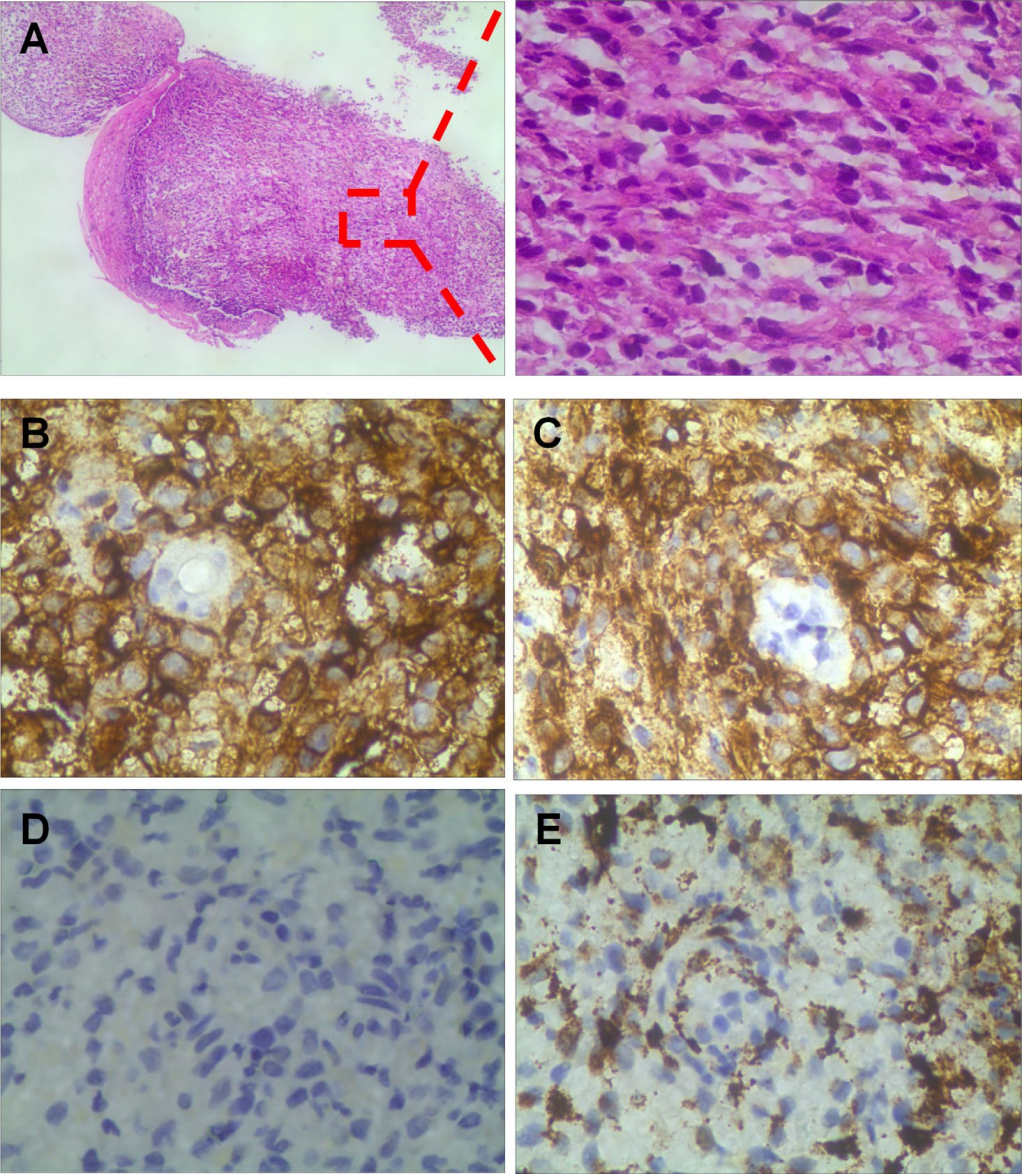
Immunophenotype isolated Langerhans cell histiocytosis of the esophagus. (A) Pathological biopsy showed abnormal proliferation of histiocytes, characterized by curled nuclei and absent nucleoli. (B) Tumor cells positive for CD1a (400×). (C) Tumor cells positive for S‐100 (400×). (D) Tumor cells positive for CD68 (400×). (E) The Ki‐67 labeling index was 30%.

Histological examination revealed a diagnosis of LCH. Pathologic biopsy showed abnormal proliferation of histiocytes with abundant cytoplasm, curled coffee bean‐like nuclei, inconspicuous nucleoli, and occasional eosinophils. Immunohistochemical features included CD1a (3+), S‐100 (3+), CD68 (+), and Ki‐67 proliferation index of 30% (Figure 3B–E). Based on these results, the patient was diagnosed with adult localized LCH.

## 3 Differential Diagnosis, Investigations, and Treatment

The patient presented with recurrent epigastric discomfort, a symptom lacking specificity. Initial evaluation necessitated differential diagnosis from common conditions including gastritis, peptic ulcer disease, and even gastrointestinal malignancies. Systematic investigations revealed no abnormalities in liver or renal function, nor evidence of common malignancies through laboratory testing. Upper gastrointestinal endoscopy identified a 5‐mm yellowish flat elevated lesion in the esophagus, and whole‐body PET/CT subsequently identified a localized esophageal lesion and metabolically active cervical lymph nodes, respectively, providing crucial diagnostic clues.

Biopsy demonstrated characteristic histiocytic proliferation and eosinophilic infiltration. Immunohistochemical staining showed strong positivity for CD1a (3+), S‐100 (3+), and CD68 (+), with a Ki‐67 proliferation index of 30%, confirming the diagnosis of localized Langerhans cell histiocytosis. Given the localized nature of the lesion, the patient successfully underwent endoscopic submucosal dissection (ESD) for complete excision. For the cervical lymph nodes, a strategy of close follow‐up was adopted. This case highlights how uncommon etiologies may underlie common symptoms, with diagnosis and management relying on multidisciplinary collaboration involving endoscopy, imaging, and pathology.

## 4 Conclusion and Results (Outcome and Follow‐Up)

The patient recovered well after endoscopic submucosal dissection and remained disease‐free during 10 months of follow‐up. This case highlights that LCH should be considered in the differential diagnosis of esophageal lesions, even in the absence of systemic involvement. During histopathological diagnosis, special attention should be paid to the differential diagnosis of LCH from common gastrointestinal malignancies such as squamous cell carcinoma. Although the prognosis for localized LCH is favorable, long‐term follow‐up is recommended to monitor for recurrence or disease progression.

## 5 Discussion

LCH is a clonal proliferative disorder driven by aberrant activation of the MAPK signaling pathway, with BRAF‐V600E mutations identified in approximately 50%–65% of cases [8, 10, 11]. While LCH can involve multiple organ systems, single‐system disease confined to a single site, as in the present case, carries an excellent prognosis.

Isolated esophageal LCH in adults is exceptionally rare. As summarized in Table 1, only five previous cases have been reported [12, 13, 14, 15]. Patient ages ranged from 42 to 63 years, with symptoms including dysphagia or epigastric discomfort when present. All reported lesions were focal, located in the mid to distal esophagus, and ranged from 0.5 to 2.0 cm in size. Treatment modalities included surgical resection (*n* = 2), endoscopic resection (*n* = 2), and observation (*n* = 1), with all patients achieving favorable outcomes during follow‐up periods ranging from 6 to 36 months. Our case represents the youngest reported patient to date and further supports endoscopic resection as a safe and effective treatment option for localized disease.

**TABLE 1:** Summary of published cases of isolated esophageal Langerhans cell histiocytosis in adults.

References	Age/Sex	Symptoms	Location	Size	Treatment	Follow‐up	Outcome
Singhi & Montgomery, 2011 [12]	42/M	Dysphagia	Distal esophagus	1.5 cm	Surgical resection	12 months	NED
Behdad & Owens, 2014 [13]	54/F	Retrosternal pain	Mid esophagus	0.8 cm	Endoscopic resection	6 months	NED
Matsuoka et al., 2021 (Case 1) [14]	63/M	Epigastric discomfort	Lower esophagus	1.2 cm	ESD	24 months	NED
Matsuoka et al., 2021 (Case 2) [14]	58/M	Asymptomatic (incidental)	Upper esophagus	0.6 cm	Observation	18 months	Stable
Shang et al., 2024 [15]	47/F	Dysphagia	Mid esophagus	2.0 cm	Surgical resection	36 months	NED
Present case	34/M	Epigastric pain	Mid esophagus	0.5 cm	ESD	10 months	NED

Abbreviations: ESD, endoscopic submucosal dissection; NED, no evidence of disease.

The diagnosis of esophageal LCH relies on histopathological examination, which demonstrates infiltration by Langerhans cells with characteristic nuclear grooves and eosinophilic cytoplasm, set against an inflammatory background [16]. Immunohistochemical positivity for CD1a and S‐100, as seen in our patient, is essential for confirming the diagnosis and distinguishing LCH from other histiocytic disorders or malignancies such as squamous cell carcinoma and melanoma. PET/CT is valuable for assessing systemic involvement, but metabolic activity in lymph nodes, as observed in this case (SUVmax = 4.4), is nonspecific and requires clinical correlation. In our patient, the absence of histopathological confirmation and the benign clinical course supported a reactive etiology for the cervical lymph nodes.

For localized single‐system LCH, current evidence supports local excision as the preferred approach, with excellent outcomes [17]. In a previous study of 10 patients with upper gastrointestinal LCH, treatment modalities included surgical resection (*n* = 2), endoscopic submucosal dissection (*n* = 2), and conservative observation (*n* = 8), all with favorable results during follow‐up [14]. These findings align with our case, where complete endoscopic resection achieved disease control without recurrence at 10 months. However, long‐term follow‐up remains essential, as 10%–20% of patients with single‐system LCH may experience disease progression or local recurrence [18].

This study has several limitations. First, as a single case report, the findings may not be generalizable. Second, molecular analysis for BRAF‐V600E and other MAPK pathway mutations was not performed; while this does not alter clinical management for completely resected localized lesions, it would have provided additional biological insight. Future studies with molecular profiling are warranted to better understand the pathogenesis of esophageal LCH.

In conclusion, this case adds to the limited literature on isolated esophageal LCH in adults and demonstrates that endoscopic resection is a safe and effective treatment option. Clinicians should maintain a high index of suspicion for LCH when encountering esophageal lesions, and histopathological evaluation with immunohistochemistry is essential for accurate diagnosis. Long‐term follow‐up is recommended to monitor for recurrence or systemic progression.

## Author Contributions

**Jingxin Ye:** conceptualization, data curation, funding acquisition, methodology, writing – review and editing. **Feng Su:** resources, supervision. **Jing Hou:** conceptualization, methodology, resources. **Mengmeng Ye:** conceptualization, methodology, software. **Zilong Ni:** investigation, methodology.

## Funding

The work was supported by Natural Science Foundation Project of Suqian Science and Technology and Technology Bureau (Grant No. K202412).

## Consent

Written informed consent was obtained from the patient.

## Conflicts of Interest

The authors declare no conflicts of interest.

## Data Availability

The data that support the findings of this study are available from the corresponding author upon reasonable request.
